# AS03-Adjuvanted, Very-Low-Dose Influenza Vaccines Induce Distinctive Immune Responses Compared to Unadjuvanted High-Dose Vaccines in BALB/c Mice

**DOI:** 10.3389/fimmu.2015.00207

**Published:** 2015-04-29

**Authors:** Karen K. Yam, Jyotsana Gupta, Kaitlin Winter, Elizabeth Allen, Angela Brewer, Édith Beaulieu, Corey P. Mallett, David S. Burt, Brian J. Ward

**Affiliations:** ^1^Department of Experimental Medicine, Research Institute of the McGill University Health Centre, Montreal, QC, Canada; ^2^GSK Vaccines, Laval, QC, Canada; ^3^Vaccine Study Centre, Research Institute of the McGill University Health Centre, Montreal, QC, Canada

**Keywords:** influenza, vaccine, AS03, adjuvant, dose-sparing

## Abstract

During the 2009–2010 influenza pandemic, an adjuvanted, dose-sparing vaccine was recommended for most Canadians. We hypothesize that differences exist in the responses to AS03-adjuvanted, low antigen (Ag) dose versus unadjuvanted, full-dose vaccines. We investigated the relationship between Ag dose and the oil-in-water emulsion Adjuvant System AS03. BALB/c mice received two IM doses of AS03_A_ or AS03_B_ with exaggerated dilutions of A/Uruguay/716/2007 H3N2 split virion vaccine Ag. Immune responses were assessed 3 weeks after the booster. Unadjuvanted “high” (3 μg) and low-dose (0.03–0.003 μg) vaccines generated similar serum antibody titers and cytokine secretion patterns in restimulated splenocytes. Compared to unadjuvanted “high-dose” vaccination, both AS03_A_ and AS03_B_-adjuvanted low-dose vaccines tended to elicit higher serum antibody titers, broader induction of cytokine secretion and generated more influenza-specific antibody secreting cells and cytokine-secreting CD4 and CD8 T cells in splenocytes. We show that varying Ag and/or AS03 dose in this influenza vaccination mouse model can strongly influence both the magnitude and pattern of the immune response elicited. These findings are highly relevant given the likelihood of expanded use of adjuvanted, dose-sparing vaccines and raise questions about the use of “standard” doses of vaccines in pre-clinical vaccine studies.

## Introduction

Vaccines are the most cost-effective method to prevent influenza virus-associated morbidity and mortality (1); however, they are also least effective in high-risk populations (1). To improve vaccine efficacy, manufacturers are increasingly turning to adjuvants. During the 2009–2010 A/California pandemic H1N1 influenza outbreak, the World Health Organization recommended the use of antigen (Ag)-sparing vaccines (2). In Canada, an Ag-sparing vaccine formulated with AS03 and 25% of the adult hemagglutinin (HA) dose in unadjuvanted seasonal influenza vaccines was selected for administration to the majority of Canadians (*Arepanrix*™; GSK Vaccines, Mississauga, ON, Canada) (3). A similar vaccine was used in Europe (*Pandemrix*™, GSK Vaccines, Rixensart, Belgium).

AS03 is an oil-in-water adjuvant consisting of squalene, alpha-tocopherol, and polysorbate-80. The greatest experience with AS03 to date has been with vaccines targeting influenza. This work demonstrated that an AS03-adjuvanted vaccine formulated with inactivated monovalent influenza A/Vietnam/1194/2004 H5N1 at low Ag dose (3.8 μg) was able to meet CHMP/FDA licensure criteria (4). There are two formulations of this adjuvant: AS03_A_ containing 11.86 mg alpha-tocopherol, 10.69 mg squalene, and 4.86 mg polysorbate-80 per 0.5 ml dose; and AS03_B_, containing 50% of each of the AS03_A_ components. Human studies have shown that vaccine formulations with AS03_A_ and AS03_B_ generate comparable short-term antibody responses (5), and that low Ag doses (3.75 or 1.9 μg) adjuvanted with AS03_A_ or AS03_B_ are non-inferior in terms of antibody production to unadjuvanted vaccines at higher Ag doses (6).

In vaccine formulations, AS03 functions to enhance the generation of Ag-specific memory B cells and polyfunctional CD4+ T cells (7, 8), induce cross-reactive antibody responses (9). In mouse studies, AS03 also induces transient innate immune responses at the site of injection, which may increase adaptive immune responses (10).

Previously, we observed that some young children who received the *Arepanrix*™ vaccine exhibited unusual avidity profiles after vaccination (11), although in HIV-positive adults, high avidity antibodies were maintained up to 6 months after immunization (12). Given the two major changes in vaccine formulation (addition of AS03 and reduced Ag dose), we wished to determine if there were differences in the immune response after immunization with unadjuvanted full-Ag dose versus AS03-adjuvanted low-dose vaccines.

Typically, dose-sparing strategies use a fixed amount of the adjuvant with varying Ag doses to identify the best formulation. We took the same approach but with exaggerated Ag dilutions with an influenza A/H3N2 split virion model Ag (A/Uruguay/716/2007) to investigate the relationship between Ag dose and AS03. We show that varying one or the other can change the immune outcomes. High- and low-dose unadjuvanted vaccines generated similar immune responses in BALB/c mice. AS03-adjuvanted vaccines were able to generate superior humoral responses and distinct cellular immune responses compared to unadjuvanted vaccine.

## Materials and Methods

### Vaccine, adjuvant, and mouse immunizations

Vaccine doses of 50 μl contained monovalent influenza A/Uruguay/716/2007 H3N2 detergent-split inactivated Ag (30 pg–3 μg HA content; GSK Vaccines, Ste-Foy, QC, Canada) and one of two variants of AS03 (GSK Vaccines, Rixensart, Belgium). AS03 is an Adjuvant System, which contains α-tocopherol and squalene in an oil-in-water emulsion. In human vaccines, AS03_A_ contains 11.86 mg α-tocopherol and AS03_B_ contains 5.93 mg α-tocopherol. For this publication, the quantities of the constituents in the murine doses of AS03_A_ and AS03_B_ were 10-fold lower than the respective human doses. Eight- to ten-week-old female BALB/c mice (Charles River Laboratories, Montreal, QC, Canada) were immunized by injection into the *gastrocnemius* muscle on days 0 and 21 (0.5 CC syringe with 28G 1/2 needle). Before each immunization, blood was collected from the lateral saphenous vein. At 3 weeks after the booster immunization, mice were sacrificed; serum and splenocytes were collected from each mouse and processed individually as described below. All procedures were carried out in accordance with guidelines of the Canadian Council on Animal Care, as approved by the Animal Care Committee of McGill University.

### Antibody titer measurement

Blood was collected in microtainer serum separator tubes (BD Biosciences, Mississauga, ON, Canada). Cleared serum samples were obtained following manufacturer’s protocol and stored at −20°C until analysis.

Hemagglutination inhibition (HAI) and microneutralization (MN) titers were measured in serum as previously described (11, 13).

ELISA protocols were optimized (14, 15) to determine HA-specific IgG concentration and avidity. Duplicate U-bottom high-binding 96-well ELISA plates (Greiner Bio-one, Frickenhausen, Germany) were coated with recombinant HA protein from A/Brisbane/10/2007 H3N2 (0.5 μg/ml) (Immune Technology Corp., New York, NY, USA) and a standard curve of mouse IgG antibodies (Sigma, St. Louis, MO, USA) in 100 mM bicarbonate/carbonate buffer at pH 9.5 [50 μl/well, overnight (O/N) at 4°C]. The A/Uruguay/716/2007 H3N2 vaccine strain used in this study is an A/Brisbane/10/2007 H3N2-like strain and shares 100% homology with each other. Before and after each step, wells were washed with PBS. Wells were blocked with 2% bovine serum albumin (BSA; Sigma) in PBS-Tween 20 (0.05%; Fisher Scientific, Ottawa, ON, Canada) (blocking buffer) (150 μl/well, 1 h at 37°C). Serum samples were diluted 1:50 in blocking buffer and added to triplicate wells of duplicate plates (50 μl/well, 1 h at 37°C); blocking buffer was added to standard curves. Next, one plate was incubated with 6M urea in PBS for 15 min at room temperature (RT), while standard curves and the second plate were incubated with blocking buffer. After washing, plates were blocked again (150 μl/well, 1 h at 37°C) and then HRP-conjugated anti-mouse total IgG antibodies (Jackson ImmunoResearch Laboratories Inc., West Grove, PA, USA) diluted 1:10,000 in blocking buffer was used (75 μl/well, 1 h at 37°C). Plates were detected with 3,3′,5,5′-tetramethyl benzidine (TMB) substrate (Millipore, Billerica, MA, USA) and stopped after 15 min with 0.5M H_2_SO_4_. Plates were read at 450 nm on an EL800 microplate reader (BioTek Instruments Inc., Winooski, VT, USA). The concentration of HA-specific IgG antibodies was determined using the standard curve included on each plate. The avidity index is calculated as (IgG concentration remaining after urea incubation)/(total IgG concentration) × 100%.

### Splenocyte isolation

Spleens were excised, collected in Hank’s balanced salt solution without calcium and magnesium (HBSS) (Wisent, St. Bruno, QC, Canada), and processed individually. Homogenous cell suspensions were prepared by passing organs through a 70 μm cell strainer (BD Biosciences, Mississauga, ON, Canada). Cells were treated with ACK buffer (0.15M NH_4_Cl, 1 mM KHCO_3_, 0.1 mM Na_2_EDTA; pH 7.2), and then washed with HBSS. Splenocytes were resuspended in RPMI supplemented with 10% fetal bovine serum (FBS), 1 mM penicillin/streptomycin (all from Wisent), and 0.5 mM β-mercaptoethanol (Sigma) (complete RPMI, cRPMI).

### Splenocyte stimulation and cell proliferation assay

Splenocytes were seeded in duplicate in 96-well U-bottom plates (BD Falcon, Mississauga, ON, Canada) at 10^6^ cells in 200 μl with cRPMI alone (unstimulated) or with A/Uruguay/716/2007 H3N2 split vaccine (2.5 μg/ml HA) in cRPMI. After 72 h at 37°C +5% CO_2_, plates were spun down (300 × *g*, 10 min at RT) and supernatant was collected and stored at −80°C until analysis. Cells were pulsed with 1 μCi/well H^3^-Thymidine (MP Biomedical, Solon, OH, USA) for an additional 18 h. After one freeze–thaw, cells were harvested on glass–fiber filters with a Tomtec harvester 96 (Tomtec Inc., Hamden, CT, USA) and H^3^-thymidine incorporation was measured by scintillation counter (Wallac Microbeta Trilux 1450 beta-counter; Wallec, Turku, Finland). Cell proliferation values were expressed as stimulation index (SI); for each mouse SI = (average Ag-stimulated cpm)/(average unstimulated cpm).

### Quantitation of cytokines in supernatant

The concentrations of 16 cytokines and chemokines (IL-1α, IL-1β, IL-2, IL-3, IL-4, IL-5, IL-6, IL-10, IL-12p70, IL-17, MCP-I, IFNγ, TNFα, MIP-1α, GM-CSF, and RANTES) in culture supernatants after 72 h stimulation *in vitro* were determined using Q-Plex Mouse Cytokine – Screen (16-plex) multiplex ELISA following the manufacturer’s guidelines (Quansys Biosciences, Logan, UT, USA). Ag-stimulated supernatant samples for each mouse were run as singlets. Unstimulated samples were pooled for each group and run as singlets.

### ELISpot assays

Influenza HA-specific antibody secreting cells (ASCs) were determined using the ELISpot^Plus^ for Mouse IgG kit (MabTech Inc., Mariemont, OH, USA) following Protocol I using biotinylated Ag. Biotinylated HA was prepared using recombinant HA protein from A/Brisbane/10/2007 H3N2 (Immune Technology Corp.). Biotinylation was performed using the EZ-Link Micro Sulfo-NHS-LC-Biotinylation kit (Thermo Scientific, Lafayette, CO, USA) following the manufacturer’s protocol. Wells were coated with mouse anti-IgG capture antibody according to the manufacturer’s protocol. Splenocytes (2.5 × 10^5^ to 10^6^ cells in 150 μl) were added to duplicate wells and incubated for 16 h at 37°C +5% CO_2_. Biotinylated HA protein (1 μg/ml), Streptavidin-alkaline phosphatase (ALP), and nitroblue tetrazolium chloride/5-bromo-4-chloro-3-indolyl phosphate (BCIP/NBT)-plus substrate were used to detect HA-specific IgG ASCs according to the manufacturer’s protocol (MabTech Inc.). Plates were read using a CTL series 3B ImmunoSpot analyzer (CTL Analyzers LLC, Cleveland, OH, USA) with ImmunoSpot 4.0.3 software supplied by the manufacturer.

To estimate numbers of memory B cells, splenocytes were stimulated for 5 days *ex vivo* to generate memory B cell-derived plasma cells before plating onto ELISpot plates as described above. The stimulation protocol was modified from (16, 17); 4 × 10^6^ splenocytes were stimulated with 2 μg/ml CpG Prototype ODN 2006 (Hycult Biotech, Plymouth Meeting, PA, USA) and 50 U/ml hIL-2 (kindly provided by Dr. Ciriaco A. Piccirillo, McGill University) in 2 ml cRPMI for 5 days. Cells were collected, washed, and seeded onto pre-coated ELISpot plates as described above. Splenocytes cultured without stimulation for 5 days were included as negative controls, and were at baseline (data not shown).

### Splenocyte stimulation, intracellular staining, and flow cytometry analysis

Protocols were modified from Moris et al. (7) to analyze influenza-specific T cell responses. Splenocytes from each mouse were seeded in 96-well U-bottom plates (BD Biosciences) (10^6^ cells in 200 μl/well) and stimulated as singlets with anti-mouse CD28 (37.51) and CD49b (HMa2) antibodies for co-stimulation (both from eBioscience, San Diego, CA, USA) at 2 μg/ml (background) or both antibodies with A/Uruguay/716/2007 H3N2 split vaccine (10 μg HA/ml) (Ag-stimulated) in cRPMI. Following 13 h at 37°C +5% CO_2_, Brefeldin A (eBioscience) was added according to manufacturer’s protocol and incubated for 5 h. As positive controls, pooled splenocytes for each group were co-stimulated with antibodies described above, and with phorbol myristate acetate (PMA) + ionomycin (2.5 and 5 μg/ml, respectively) and Brefeldin A for 5 h. Control samples were stained and analyzed as described below (data not shown). After incubation, cells were transferred to V-bottom plates (BD Biosciences) for FC staining.

Antibodies used, the staining procedure and gating strategy are described in Figure S1 in Supplementary Material. Briefly, cells were stained for CD3, CD4, and CD8 on the surface and intracellularly for IL-2, IL-5, IFNγ, and TNFα. Results are expressed as the percentage of CD4+ and CD8+ T cells producing all combinations of the cytokines tested. Background (cells stimulated with anti-CD28 and anti-CD49d antibodies only) was subtracted from Ag-stimulated values (cells stimulated with antibodies and A/Uruguay H3N2 split vaccine).

### Statistical analysis

For serum antibody titers and avidity, estimates of the geometric mean ratios between groups and their 95% confidence intervals (CI) were obtained using back-transformation on log10 values. All experiments were analyzed by one-way analysis of variance (ANOVA) followed by Tukey post-test to compare all possible pairs of groups. Analyses were performed using GraphPad Prism 5.0 software.

## Results

### Adjuvanted, low-dose vaccines generate superior antibody responses than unadjuvated vaccines

**Figure 1:**
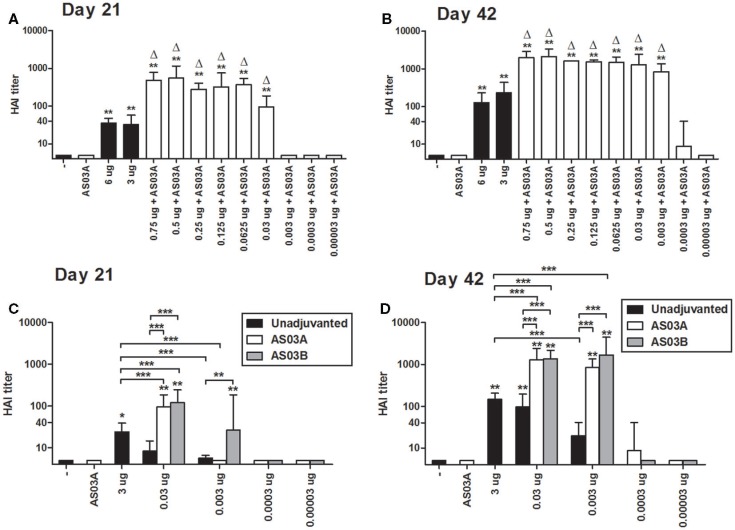
**Hemagglutination inhibition (HAI) titers in BALB/c mice after one or two immunizations of unadjuvanted A/Uruguay/716/2007 H3N2 split vaccine or AS03-adjuvanted dose-sparing vaccines**. Mice were immunized intramuscularly on days 0 and 21, and sera from individual mice were analyzed. HAI titers of mice immunized with AS03_A_-adjuvanted vaccines on days 21 **(A)** and 42 **(B)**. HAI titers of mice immunized with AS03_A_ or AS03_B_-adjuvanted vaccines on days 21 **(C)** and 42 **(D)**. On top of bars, * indicates a significant increase (*P* < 0.05) to negative “−” group and ** indicates a significant increase (*P* < 0.05) to both negative “−” and AS03_A_ only control groups. For **(A,B)**, delta symbols (Δ) on top of bars indicate a significant increase (*P* < 0.05) compared to mice immunized with 3 μg vaccine only. For **(C,D)** significant differences between groups are denoted by brackets; *P < 0.05; **P < 0.01; ***P < 0.001. For **(A,B)** data represent 4–12 individual mice per group combined from two independent studies. For **(C,D)**, data represent 4–17 or 4–25 mice per group combined from three or four independent studies, respectively. Geometric means and 95% confidence intervals are shown.

Two formulations of unadjuvanted high-Ag dose vaccines (3 and 6 μg/dose) were selected based on previous studies (18). After one or two immunizations, mice produced comparable HAI titers, which were significantly higher than the control groups (Figures 1A,B).

Based on the *Arepanrix*™ vaccine and other mouse studies, we selected 0.75 μg as the starting point for our low Ag formulations (3, 18). After one immunization, mice vaccinated with 0.75 μg + AS03_A_ produced significantly higher HAI titers than either high-dose unadjuvanted vaccine (3 or 6 μg/dose; Figure 1A). Progressively lower doses of Ag administered with AS03_A_ were able to generate detectable HAI titers after one immunization, but titers began to fall off at doses below 0.06 μg (Figure 1A).

Booster immunization increased HAI titers overall (Figure 1B). Mice immunized with low Ag dose (0.003–0.75 μg/dose) with AS03_A_ produced significantly higher HAI titers than 3 μg without adjuvant (Figure 1B). Interestingly, although no response was detected after one dose, titers increased remarkably in the 0.003 μg + AS03_A_ group after boosting (Figure 1B).

We next compared AS03_A_ with half the amount of adjuvant (AS03_B_) to unadjuvanted formulations. After a single immunization, unadjuvanted low-dose (0.03–0.003 μg) vaccines elicited low but detectable HAI titers (Figure 1C). Generally, use of the adjuvant increased antibody titers. AS03_B_ functioned as efficiently as AS03_A_ with 0.03 μg Ag, but at the lower dose (0.003 μg), AS03_B_ generated a better response than AS03_A_ in terms of HAI antibodies (Figure 1C). After booster immunization, 0.03 or 0.003 μg Ag with AS03_A_ or AS03_B_ generated similar HAI titers, which were higher than unadjuvanted vaccine (Figure 1D). Unadjuvanted low-dose vaccines tended to generate lower titers than the unadjuvanted high-dose formulation.

We were unable to reproducibly detect HAI titers after two immunizations at Ag doses of ≤0.0003 μg with AS03_A_ or AS03_B_ in our mouse model (Figures 1C,D). These very low Ag doses also failed to generate detectable immune responses by ELISA, MN assays, ELISpot, and lymphoproliferation in restimulated splenocytes (data not shown). Therefore, we focused on low Ag doses of 0.03 and 0.003 μg in our subsequent studies.

**Figure 2:**
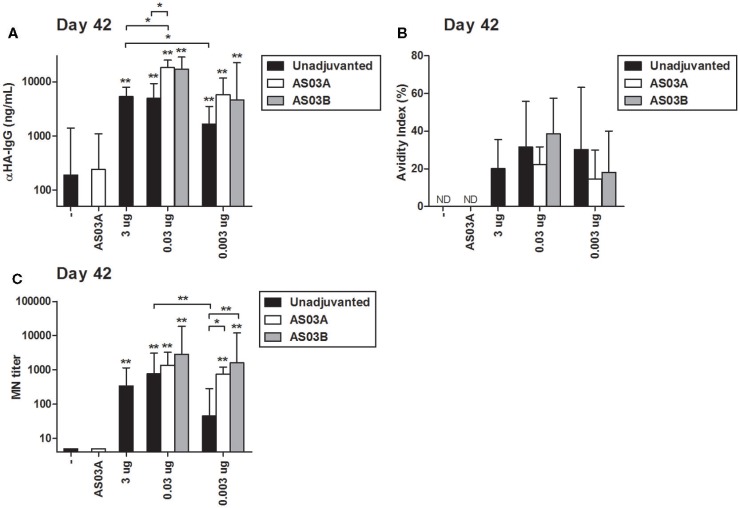
**Influenza HA-specific IgG concentrations, antibody avidity, and microneutralization (MN) titers in BALB/c mice after one or two immunizations of unadjuvanted A/Uruguay/716/2007 H3N2 split vaccine or AS03-adjuvanted dose-sparing vaccines**. Mice were immunized intramuscularly on days 0 and 21, and sera from individual mice were analyzed. Anti-HA IgG concentrations **(A)** and antibody avidity **(B)** measured by ELISA, and MN titers **(C)** were determined on day 42. On top of bars, * indicates a significant increase (*P* < 0.05) to negative “−” group and ** indicates a significant increase (*P* < 0.05) to both negative “−” and AS03_A_ only control groups. Significant differences between groups are denoted by brackets; *P < 0.05; **P < 0.01; ***P < 0.001. For **(A,B)**, data represent 4–10 mice per group combined from three independent studies. For **(C)**, data represent 4–12 mice per group combined from three independent studies. Geometric means and 95% confidence intervals are shown. ND, not detected.

Similar to the HAI results, two immunizations of AS03-adjuvanted, low-dose vaccines tended to induce higher concentrations of influenza HA-specific IgG by ELISA than unadjuvanted vaccine (Figure 2A). The strength of antibody binding (avidity) was not significantly changed in response to Ag dose or use of AS03 adjuvant (Figure 2B). After two immunizations with 0.03 μg + AS03_A/B_ or 0.003 μg + AS03_A/B_, MN titers were equivalent or superior to those observed with unadjuvanted formulations (Figure 2C).

These data demonstrate that strong serum antibody responses can be elicited in BALB/c mice with 100- or 1000-fold lower Ag than a high-Ag dose and that AS03 can increase serological responses even at very low Ag doses.

### Adjuvanted low-dose and unadjuvanted vaccines generate distinct antigen-specific cytokine profiles

**Figure 3:**
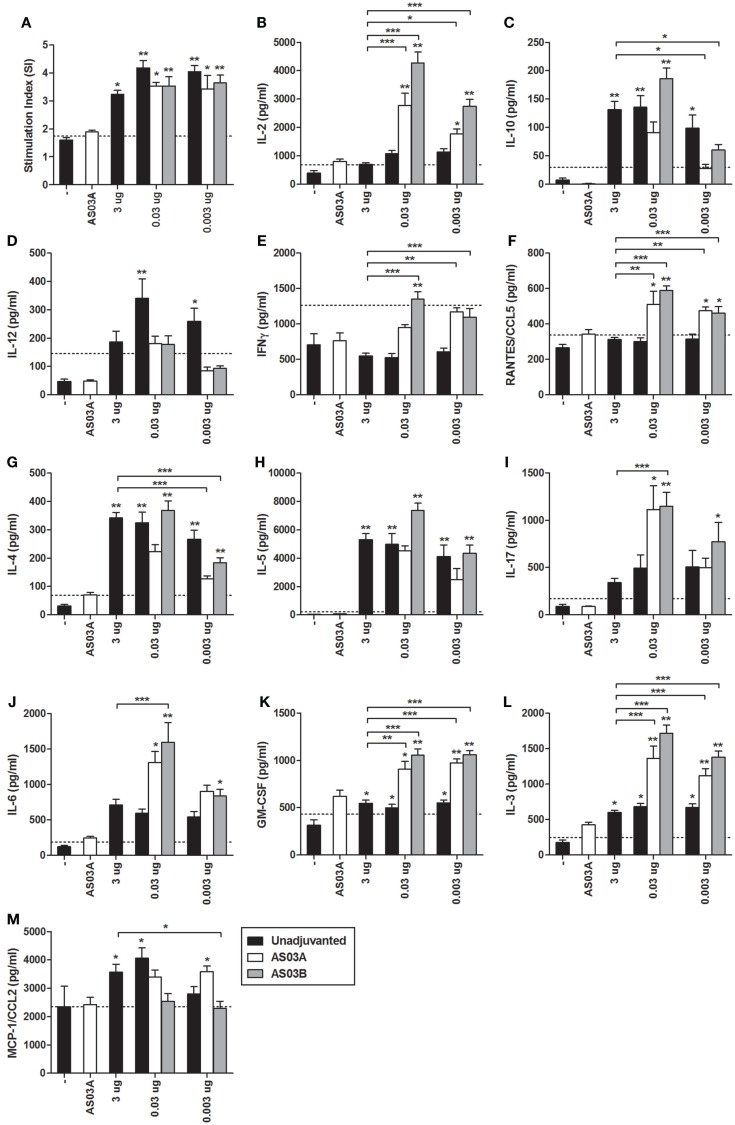
**Influenza-specific splenocyte proliferation and cytokine production after two immunizations of unadjuvanted A/Uruguay/716/2007 H3N2 split vaccine or AS03-adjuvanted dose-sparing vaccines**. BALB/c mice were immunized intramuscularly on days 0 and 21, and splenocytes were isolated from individual mice on day 42. Splenocytes were stimulated *ex vivo* with media (unstimulated background) or with A/Uruguay H3N2 split vaccine. Culture supernatant was collected after 72 h and cells were pulsed with H^3^-Thymidine for an additional 18 h. Cell proliferation **(A)** is shown as a stimulation index (SI). The line represents the mean +2 SDs of the negative control group. The concentrations of cytokines and chemokines in culture supernatants were determined using Q-Plex Mouse Cytokine – Screen (16-plex) multiplex ELISA and are summarized in Table 1: **(B)** IL-2, **(C)** IL-10, **(D)** IL-12, **(E)** IFNγ, **(F)** RANTES/CCL5, **(G)** IL-4, **(H)** IL-5, **(I)** IL-17, **(J)** IL-6, **(K)** GM-CSF, **(L)** IL-3, **(M)** MCP-1/CCL2. The line represents the mean +2 SDs of the cytokine concentration of unstimulated samples for all groups. On top of bars, * indicates a significant increase (*P* < 0.05) to negative “−” group and ** indicates a significant increase (*P* < 0.05) to both negative “−” and AS03_A_ only control groups. Significant differences between different groups are denoted by brackets; *P < 0.05; **P < 0.01; ***P < 0.001. Data represent mean and SEs of 3–19 mice per group combined from three independent studies.

All Ag-containing vaccines elicited detectable levels of Ag-specific lymphoproliferation in *ex vivo* stimulated splenocytes (Figure 3A). No statistical differences in proliferation were observed between any of the formulations so the immune microenvironment was further assessed by measuring cytokine/chemokine concentrations in culture supernatants.

**Table 1:** **Summary of influenza-specific cytokine responses in the supernatants of restimulated splenocytes after two immunizations of unadjuvanted A/Uruguay/716/2007 H3N2 split vaccine or AS03-adjuvanted dose-sparing vaccines**.

Category	Cytokine	High-Ag dose	Low Ag dose	
		Unadjuvanted^a^	Unadjuvanted^a^^,^^b^	Adjuvanted^a^^,^^b^
		3 μg	0.03 or 0.003 μg	0.03 or 0.003 μg + AS03_A/B_
T cell proliferation	IL-2			++
Anti-inflammatory	IL-10	++	++	++
Th1	IL-12		++	
	IFNγ			++
	RANTES/CCL5			++
Th2	IL-4	++	++	++
	IL-5	++	++	++
Th17	IL-17			++
	IL-6			++
Growth promoting and chemokines	GM-CSF	+	+	++
	IL-3	+	+	++
	MCP-1/CCL3	+	+	+

*^a^The “+” sign indicates a significant increase (*P* < 0.05) to negative “−” group and “++” indicates a significant increase (*P* < 0.05) to both negative “−” and AS03_A_ only control groups*.

*^b^The “+” or “++” signs indicate a significant difference by 0.03 and/or 0.003 μg Ag dose with or without AS03*.

Unadjuvanted vaccine at all Ag doses generated similar levels of all cytokines tested (Figure 3). However, adjuvanted low-dose vaccines generated very different influenza-specific cytokine milieus compared to unadjuvanted formulations. Some cytokines/chemokines, such as IL-4, IL-5, IL-10, and MCP-1/CCL2 were produced at similar levels in all groups (Figure 3), while others such as IL-2, IL-3, IL-6, IFNγ, RANTES/CCL5, IL-17, and GM-CSF were more strongly induced by the low-dose adjuvanted vaccines (Figure 3). The cytokine/chemokine responses are summarized in Table 1. AS03 tended to change the balance of cytokine responses by activating different arms of the immune response (Th1, Th2, Th17, and growth promoting cytokines) (Table 1). In comparison, all unadjuvanted groups produced similar cytokine profiles. No significant differences in the levels of IL-1α, IL-1β, TNFα, and MIP-1α were observed (data not shown).

We focused our next studies on immune responses at the cellular level. Given that no significant differences in proliferation or cytokine production were observed between unadjuvanted vaccines at different Ag doses, we focused our analysis on unadjuvanted high-dose versus AS03-adjuvanted low-dose formulations.

### Adjuvanted low-dose vaccines generate more ascs than high-dose unadjuvated vaccine

**Figure 4:**
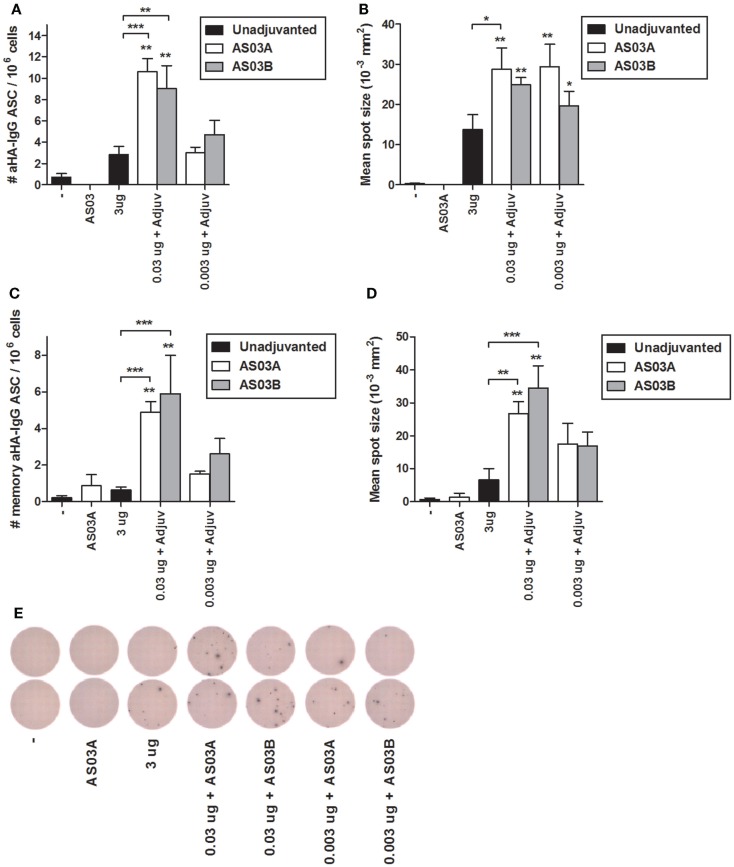
**Generation of influenza HA-specific antibody secreting cells (ASCs) and memory B cell-derived ASCs after two immunizations of unadjuvanted A/Uruguay/716/2007 H3N2 split vaccine or AS03-adjuvanted dose-sparing vaccines**. BALB/c mice were immunized intramuscularly on days 0 and 21, and splenocytes were isolated from individual mice on day 42. The number **(A)** and mean spot size **(B)** of HA-specific IgG antibody secreting cells (ASCs) were determined by ELISpot. Following *ex vivo* stimulation, the number **(C)** and mean spot size **(D)** of memory B cell-derived HA-specific IgG ASCs were determined by ELISpot. Representative ELISpot wells are shown in **(E)**. On top of bars, * indicates a significant increase (*P* < 0.05) to negative “−” group and ** indicates a significant increase (*P* < 0.05) to both negative “−” and AS03_A_ only control groups. Significant differences between groups are denoted by brackets; *P < 0.05; **P < 0.01; ***P < 0.001. Data represent mean and SEs of 4–12 mice per group combined from two independent studies.

ELISpots were used to enumerate influenza-HA specific IgG ASCs in splenocytes (i.e., plasma cells), and memory B cell-derived plasma cells (i.e., memory ASCs) following *in vitro* differentiation. Low levels of ASCs and memory ASCs were detectable in mice immunized with unadjuvanted vaccine (Figures 4A,C,E). Mice immunized with 0.03 μg + AS03_A/B_ had higher numbers of ASCs (Figures 4A,E) and memory ASCs (Figure 4C). At the lowest Ag dose (0.003 μg + AS03_A/B_), the ASC (Figures 4A,E) and memory ASC response (Figure 4C) were comparable to that seen in mice receiving unadjuvanted vaccine. The mean spot size in these assays is correlated with the amount of antibody secreted. The adjuvanted low-dose groups tended to secret more antibodies per ASC or memory ASC than unadjuvanted vaccine (Figures 4B,D). These data suggest the adjuvanted low-dose formulations induced greater numbers of influenza-specific plasma cells and potentially memory B cell-derived ASCs that secreted more antibody per cell compared to the high-dose unadjuvanted vaccine.

### AS03_B_-adjuvanted low-dose vaccines produce more influenza-specific CD4+ and CD8+ T cells

**Figure 5:**
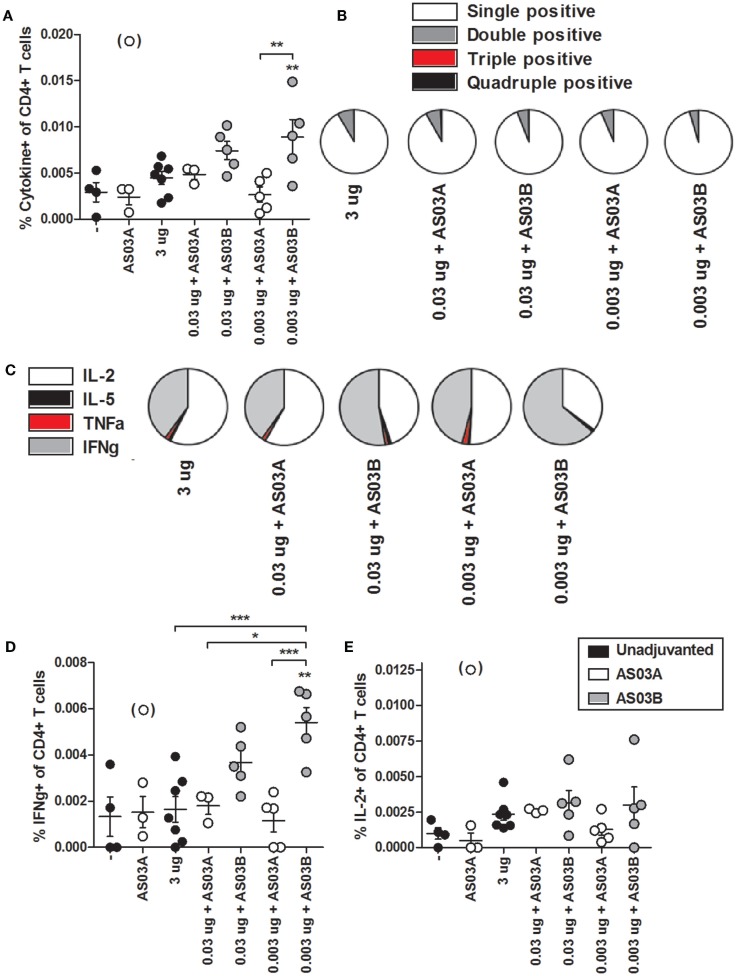
**Influenza-specific cytokine-producing CD4+ T cells in splenocytes after two immunizations of unadjuvanted A/Uruguay/716/2007 H3N2 split vaccine or AS03-adjuvanted dose-sparing vaccines**. BALB/c mice were immunized intramuscularly on days 0 and 21, and splenocytes were isolated from individual mice on day 42. Splenocytes were stimulated *ex vivo* with A/Uruguay H3N2 split vaccine and co-stimulatory antibodies, then analyzed by flow cytometry for CD4+ T cells that produced a combination of IL-2, IL-5, IFNγ, or TNFα cytokines. The gating strategy is described in Figure S2 in Supplementary Material and representative dot plots are shown in Figure S3 in Supplementary Material. The percentage of total responding CD4+ T cells that expressed any combination of the four cytokines **(A)**. The distribution of the number of individual cytokines produced by total responding CD4+ T cells **(B)**. The distribution of the specific cytokines produced by single-positive CD4+ T cells **(C)**. The percentage of CD4+ T cells that were single positive for IFNγ **(D)** or IL-2 **(E)**. On top of groups, ** indicates a significant increase (*P* < 0.05) to both negative “−” and AS03_A_ only control groups. Significant differences between groups are denoted by brackets; *P < 0.05; **P < 0.01; ***P < 0.001. Data represent mean and SEs of 3–5 mice per group. An outlier in the AS03_A_ only control group (denoted in brackets) was beyond the mean +3 SDs of the remainder of the group, and was omitted from analysis.

**Figure 6:**
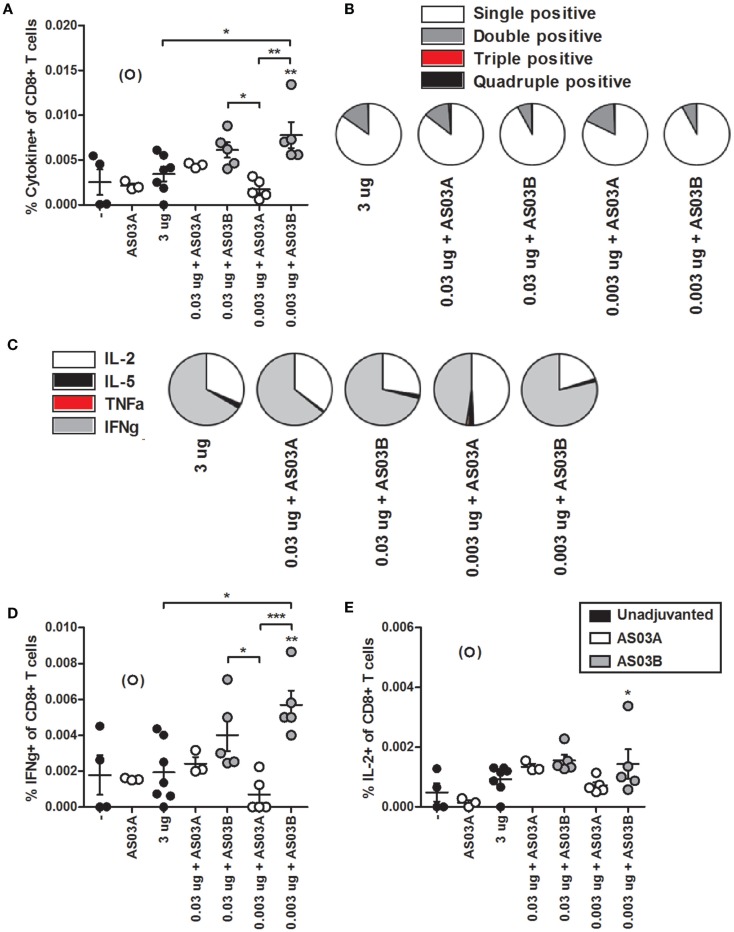
**Influenza-specific cytokine-producing CD8+ T cells in splenocytes after two immunizations of unadjuvanted A/Uruguay/716/2007 H3N2 split vaccine or AS03-adjuvanted dose-sparing vaccines**. BALB/c mice were immunized intramuscularly on days 0 and 21, and splenocytes were isolated from individual mice on day 42. Splenocytes were stimulated *ex vivo* with A/Uruguay H3N2 split vaccine and co-stimulatory antibodies, then analyzed by flow cytometry for CD8+ T cells that produced a combination of IL-2, IL-5, IFNγ, or TNFα cytokines. The gating strategy is described in Figure S2 in Supplementary Material and representative dot plots are shown in Figure S4 in Supplementary Material. The percentage of total responding CD8+ T cells that expressed any combination of the four cytokines **(A)**. The distribution of the number of individual cytokines produced by total responding CD8+ T cells **(B)**. The distribution of the specific cytokines produced by single-positive CD8+ T cells **(C)**. The percentage of CD8+ T cells that were single positive for IFNγ **(D)** or IL-2 **(E)**. On top of groups, ** indicates a significant increase (*P* < 0.05) to both negative “−” and AS03_A_ only control groups. Significant differences between groups are denoted by brackets; *P < 0.05; **P < 0.01; ***P < 0.001. Data represent mean and SEs of 3–5 mice per group. An outlier in the AS03_A_ only control group (denoted in brackets) was beyond the mean +3 SDs of the remainder of the group, and was omitted from analysis.

We investigated influenza-specific cytokine production in splenocytes by examining CD4+ or CD8+ T cells that produced IL-2, IL-5, TNFα, and IFNγ cytokines by flow cytometry (FC) (Figure S1 in Supplementary Material). Immunization with 0.003 μg + AS03_B_ generated the highest percentage of Ag-specific CD4+ (Figure 5A; Figure S2 in Supplementary Material) and CD8+ (Figure 6A; Figure S3 in Supplementary Material) T cells expressing any combination of the four cytokines tested. Generally, formulations with AS03_B_ tended to generate more cytokine-producing cells than with AS03_A_ (Figures 5A and 6A).

Ag-specific T cells can be categorized according to the number of cytokines they produced. Most CD4+ and CD8+ T cells were single positive for one of the four cytokines (Figures 5B and 6B). With all formulations, over 90% of cytokine-producing CD4+ T cells secreted a single cytokine (Figure 5B). For CD8+ T cells, AS03_A_-adjuvanted low-dose vaccines induced similar levels of poly-functional T cells (expressing two or more cytokines) as high-dose vaccine, which tended to be higher than that observed with AS03_B_-adjuvanted formulations (Figure 6B).

Ag-specific, single positive T cells can be further categorized according to the cytokine produced. Most single positive CD4+ T cells in the high-dose unadjuvanted and AS03_A_-adjuvanted low-dose groups expressed IL-2, whereas the AS03_B_-adjuvanted groups produced more IFNγ-secreting cells (Figure 5C). In all groups except 0.003 μg + AS03_A_, most single positive CD8+ T cells expressed IFNγ, followed by a smaller percentage of IL-2-secreting cells (Figure 6C). Interestingly, mice given 0.003 μg + AS03_A_ tended to generate relatively equal proportions of IL-2 and IFNγ expressing CD8+ T cells (Figure 6D), although this group tended to generate fewer total responding cells (Figure 6A). Compared to high-dose vaccine, the 0.003 μg + AS03_B_ recipients had significantly higher percentages of CD4+ and CD8+ T cells single positive for IFNγ (Figures 5D and 6D). There were no significant differences in single positive IL-2 (Figures 5E and 6E), IL-5, or TNFα (data not shown) producing cells or any combination of double or triple positive cells, and no Ag-specific, quadruple positive cells were observed (data not shown).

## Discussion

We demonstrate that use of AS03 can markedly change both the magnitude and pattern of vaccine-induced humoral and cellular immune responses in mice. Nanogram quantities of unadjuvanted vaccine were sufficient to induce Ag-specific immune responses that were, in some respects, comparable to those induced by high-dose vaccine. However, in the presence of AS03, even very low-dose (0.03–0.003 μg/dose) formulations elicited superior humoral and distinct cellular immune responses compared to unadjuvanted vaccine. Given that cell-mediated immunity is increasingly recognized to be important in protecting against influenza viral infection (19), these results suggest that over-reliance on serum antibody responses may not identify optimal vaccine formulations.

This is the first detailed pre-clinical investigation of the humoral and cellular immune responses to extreme dose-sparing with AS03. As little as 3 ng (0.003 μg) of A/Uruguay H3N2 model Ag was sufficient to induce excellent responses with or without AS03. Our findings are similar to those recently reported, which found that 0.03–0.04 μg of influenza vaccines intramuscularly injected with various adjuvants could induce Ag-specific humoral and cellular immune responses, and protect against viral infection (20, 21). In contrast to these studies, we varied both Ag and adjuvant dose and performed detailed analyses of both B and T cell responses following vaccination.

The IgG avidity result is particularly surprising since the development of high avidity antibodies is thought to require both sufficient time and presence of Ag (22). Antibodies with comparable avidity were generated with high- and low-dose Ag with or without adjuvant. This was unexpected and is possibly a testament to the remarkable efficiency of the BALB/c mouse immune system or the immunogenicity of the A/Uruguay H3N2 split virion Ag used. Future studies could use different mouse strains to further investigate antibody avidity. In contrast, human trials with a similar oil-in-water adjuvant, MF59 and A/California/07/2009 H1N1 split-virus Ag in a dose-sparing formulation (7.5 μg + MF59) increased antibody avidity compared to standard unadjuvanted vaccine (15 μg) (23, 24). Another consideration is that relatively massive Ag doses are administered to mice in most pre-clinical vaccine studies. On a body-weight basis, our 0.003 μg dose in a 20 g mouse translates into an ~10 μg dose in a 70 kg human, which is close to the standard influenza vaccine dosage per strain. Our data raise the question of whether or not the doses routinely used in pre-clinical studies should be re-evaluated.

The superior performance of the adjuvanted, low-dose formulations in the induction of ASCs that secrete more antibodies per cell was intriguing. The presence of greater numbers of highly active plasma cells likely accounts for the higher serum HAI and ELISA antibody titers observed in the 0.03 μg + AS03A/B groups, although MN titers were not similarly elevated. Surprisingly, unadjuvanted and the lowest Ag dose groups (0.003 μg + AS03A/B) generated very low numbers (close to background) of ASCs and memory ASCs in the spleen, but exhibited robust serum antibody titers. It would be interesting to investigate the presence of ASCs in other compartments (e.g., bone marrow) since plasma cells and memory B cells eventually track to the bone marrow late in the immune response (22). Differences in cell trafficking might therefore account for the seeming discrepancy between the low numbers of ASCs, but high serum antibody titers. The maintenance of long-term humoral memory is influenced by several factors including the presence of sufficient Ag (25). Indeed in our study, splenocytes from the lowest dose groups (0.003 μg + AS03_A/B_) had fewer ASCs and memory ASCs than groups immunized with 0.03 μg suggesting that Ag dose plays a role in our model in the generation of plasma and memory B cells. There were, however, no major differences in the level of antibody secretion per cell in the two adjuvanted low-dose groups.

Unadjuvanted and low-dose adjuvanted vaccines also induced distinct cytokine profiles in restimulated splenocytes. Compared to adjuvanted, low-dose vaccines, the unadjuvanted groups tended to produce higher levels of Th2 cytokines such as IL-4 and IL-5, as well as IL-10 that can promote B cell proliferation. However, serum antibody levels and the number of ASCs in the unadjuvanted groups were not higher than those seen in the adjuvanted, low-dose groups. Together, these observations suggest a Th2-biased response with ineffective antibody production. This apparent paradox might be explained by the activation of a broader range of cytokines in the adjuvanted vaccine groups leading to greater overall vaccine-specific responses. For example, the splenocytes of AS03-containing groups produced higher levels of IL-2 and IL-6 thereby suggesting activation of more T cells and greater differentiation of follicular helper T cells, respectively (26, 27). Furthermore, IL-6 also plays a role in the maturation of B cells into ASCs. The AS03-adjuvanted formulations also induced higher levels of IFNγ and RANTES/CCL5, the latter acting through the CCR5 receptor to promote development of IFNγ-producing Th1 cells (26). Surprisingly, the unadjuvanted low-dose vaccine tended to induce the highest level of IL-12, a Th1-promoting cytokine, although this difference did not reach statistical significance. Therefore, in our mouse model, both the unadjuvanted and adjuvanted formulations had the potential to induce cytokines/chemokines associated with Th1-type responses. Finally, only the adjuvanted formulations were found to induce Th17 type (IL-17 and IL-6) and growth promoting (GM-CSF and IL-3) cytokines. In summary, we found that the unadjuvanted vaccines could induce both Th1- and Th2-type responses, while AS03-adjuvanted vaccines induced Th1, Th2, Th17, and growth promoting cytokine/chemokine production in the restimulated splenocytes. Activation of this broad range of cytokines/chemokines by the adjuvanted vaccines likely contributed to the stronger Ag-specific immune responses generated.

Historically, little attention was paid to adjuvant dose except in the context of adverse events, with greater adjuvant doses tending to cause more reactions (28, 29). Little consideration was given to the idea that different doses of adjuvant might alter the pattern of the immune response induced. In a single clinical trial that varied the doses of both MF59 (full, half, or quarter) and trivalent inactivated influenza vaccine (15 or 30 μg) in elderly subjects, Della Cioppa et al. found that more adjuvant tended to induce higher HAI titers but had no effect on the Ag-specific CD4 T cell response (30). In our model, AS03_A_ and AS03_B_ (full- and half-dose, respectively) induced similar serum antibody profiles. However, the AS03_B_-adjuvanted formulations tended to produce higher levels of most of the cytokines/chemokines measured compared to AS03_A_. Formulations with AS03_B_ (especially the 0.003 μg/dose) also generated more influenza-specific CD4+ and CD8+ T cells than the unadjuvanted or AS03_A_-adjuvanted vaccines, although these cells were primarily single positive for IFNγ. However, we found that the low-dose vaccines formulated with AS03_A_ tended to generate more influenza-specific poly-functional CD8+ T cells compared to AS03_B_. Poly-functional T cells generally express higher levels of cytokines per cell and are considered to be functionally superior to single-cytokine-producing cells (31). Additional studies are needed to determine the functional significance of the mono-functional versus poly-functional T cells induced by these different formulations in our vaccine model.

In human studies of PBMCs isolated after AS03-adjuvanted vaccine administration, an increase in Ag-specific CD4+ T cells is usually observed in the absence of increasing CD8+ T cells responses (7, 32, 33). In contrast, we observed both CD4+ and CD8+ T cell responses similar to the findings of other mouse studies. For example, studies from the Boivin laboratory showed that two immunizations of AS03-adjuvanted influenza vaccine (at 3 μg/dose) produced detectable Ag-specific CD4+ and CD8+ T cells, which tended to be greater than responses in control groups, although statistical differences were not observed (18, 34). We found that reductions in Ag dose with the same dose of AS03 could markedly increase T cell responses.

Clearly, we show that greater attention should be paid to the balance of Ag and adjuvant in vaccine formulations to fully understand vaccine efficacy. However, given the known differences in T cell responses in humans versus mice, our observations in the very low-dose mouse model may not be predictive of responses in humans. The majority of adult humans have been previously exposed to various strains of influenza, which is unlike the immunologically naïve mice in our studies. In human studies with AS03, vaccine-specific CD8+ T cells were detected at all timepoints including pre-immunization, but vaccination failed to significantly increase the Ag-specific CD8 responses (32, 33). In naïve mice, we observe an increase in Ag-specific CD4+ and CD8+ T cell responses after vaccination. Indeed, the two-dose vaccination schedule that we use in our study is the same as that used in immunologically naïve infants. As a result, our observations may be more relevant to this population, rather than the response in primed adults who mainly receive a single dose. Finally, AS03 is thought to function primarily through the induction of cytokines (low level inflammation) at the site of injection (10) but the mouse and human inflammatory responses can be very different as was recently demonstrated in a comprehensive transcriptomic analysis (35). Therefore, the broad activation of cytokines we observed in groups given AS03-adjuvanted formulations may also be specific to our mouse model.

The current study has additional limitations; for example, we have not yet tested whether or not the different immune response patterns correlate with protection from influenza challenge. These studies are currently underway. The A/Uruguay H3N2 split virion Ag used in this study is also relatively more immunogenic in mice than Ag prepared from other influenza strains (unpublished observations). Therefore, our results may not extrapolate to other vaccine formulations and additional studies are warranted to test this. Although Ag doses in mouse influenza vaccine studies typically range from 3 to 15 μg, we acknowledge that our selection of 3 μg as the high-dose vaccine formulation was entirely arbitrary. As noted above, our 3 μg dose does not correspond well with the 15 μg Ag dose routinely used in human vaccines on a body-weight basis. Similarly, our selection of 25 μl/12.5 μl of AS03_A/B_ was based upon previous mouse studies (18, 36) but is also arbitrary and is much larger (350×) than the corresponding dose (by volume) used in humans for intramuscular injection (250 μl) on a body-weight basis (3).

In the event of an influenza pandemic, there will be great pressure to deliver the largest number of vaccine doses as quickly as possible. Our data suggest that both Ag and adjuvant-sparing strategies may make important contributions to optimization efforts from both immunologic and economic standpoints. We show that differences in both influenza Ag and adjuvant dose can significantly alter the immune response pattern following vaccination; findings that may be very relevant to the development of better vaccines. These observations also raise important questions about the use of “standard” doses of both Ag and adjuvants in pre-clinical vaccine studies in mice.

## Author Contributions

KY and BW designed the study and experiments. KY, JG, KW, EA, and AB performed experiments under the supervision of KY. KY and BW analyzed the data and wrote the paper with input from EB, CPM, and DB.

## Conflict of Interest Statement

Édith Beaulieu, Corey P. Mallett, and David S. Burt are employees of the GSK group of companies. The remaining authors declare no commercial or financial conflict of interest.

## Supplementary Material

The Supplementary Material for this article can be found online at http://journal.frontiersin.org/article/10.3389/fimmu.2015.00207
