# Supplementary material for: AS03-Adjuvanted, Very-Low-Dose Influenza Vaccines Induce Distinctive Immune Responses Compared to Unadjuvanted High-Dose Vaccines in BALB/c Mice
Source: Front Immunol. 2015 Apr 29;6:207. doi: 10.3389/fimmu.2015.00207 (PMC4413846; doi:10.3389/fimmu.2015.00207)

AS03-adjuvanted, very-low-dose influenza vaccines induce distinctive immune responses compared to unadjuvanted high-dose vaccines in BALB/c mice

Running title: AS03-adjuvanted low-dose influenza vaccines

Karen K. Yam[1], Jyotsana Gupta[1], Kaitlin Winter[1], Elizabeth Allen[1], Angela Brewer[1], Édith Beaulieu[3], Corey P. Mallett[3], David S. Burt[3] and Brian J. Ward[1,2,*]

[1]Department of Experimental Medicine, Research Institute of the McGill University Health Centre, Montreal, QC, Canada

[2]Vaccine Study Centre, Research Institute of the McGill University Health Centre, Montreal, QC, Canada

[3]GSK Vaccines, Laval, QC, Canada

Supplementary Materials

Figure S1

Flow cytometry antibodies uses, staining procedure and gating strategy.

Splenocytes of immunized mice were stimulated *ex vivo* with A/Uruguay H3N2 split vaccine and co-stimulatory antibodies, then analysed by flow cytometry. Antibodies used were as follows: Mouse BD Fc Block (2.4G2), V500 anti-mouse CD4 (RM4-5), Peridinin chlorophyll protein complex-Cyanine 5.5 (PerCP-Cy5.5)-labelled anti-mouse CD8a (53-6.7), Phycoerythrin (PE)-labelled anti-mouse IFNγ (XMG1.2) and Allophycocyanin (APE)-labelled anti-mouse/anti-human IL-5 (TRFK5) (all from BD Biosciences); fixable viability dye eFluor780, Fluorescein-isothiocyanate (FITC)-labelled anti-mouse CD3e (145-2C11), Phycoerythrin-Cyanine 7 (PE-Cy7)-labelled anti-mouse IL-2 (JES6-5H4), and eFluor450 anti-mouse TNFα (MP6-XT22) (all from eBioscience, San Diego, CA).

Cells were stained with fixable viability dye eFluor 780 following manufacturer's protocol, then incubated with Mouse BD Fc Block (1 μl/sample) for 10 mins, and surface stained with anti-CD3e, anti-CD4, and anti-CD8a antibodies (each at 0.5 μl/sample) for 20 mins on ice. After washing, cells were resuspended in 100 μl IC Fixation buffer (eBioscience) and store at 4 $^{\circ}$C O/N. Cells were permeabilized with Permeabilization buffer (eBioscience) and then intracellularly stained with anti-IL-2, anti-IL-5, anti- IFNγ and anti-TNFα (each at 1 μl/sample) for 45 mins on ice. After washing, data was acquired on a FACSCanto II flow cytometer using FACSDiva software (Becton Dickinson, Mountain View, CA). Singly-stained compensation controls were prepared with OneComp eBeads (eBioscience) for all antibodies with the exception of V500 anti-mouse CD4 and fixable viability dye eFluor 780, which were prepared with splenocytes. Data analysis and automatic compensation calculations were performed using FlowJo software (Tree Star, Ashland, OR).

The flow cytometry gating strategy for Figures 6 and 7 is shown. We analysed CD4+ and CD8+ T cells that produce a combination of IL-2, IL-5, IFNγ, and TNFα cytokines.

Supplemental Figure 1

Flow cytometry gating strategy

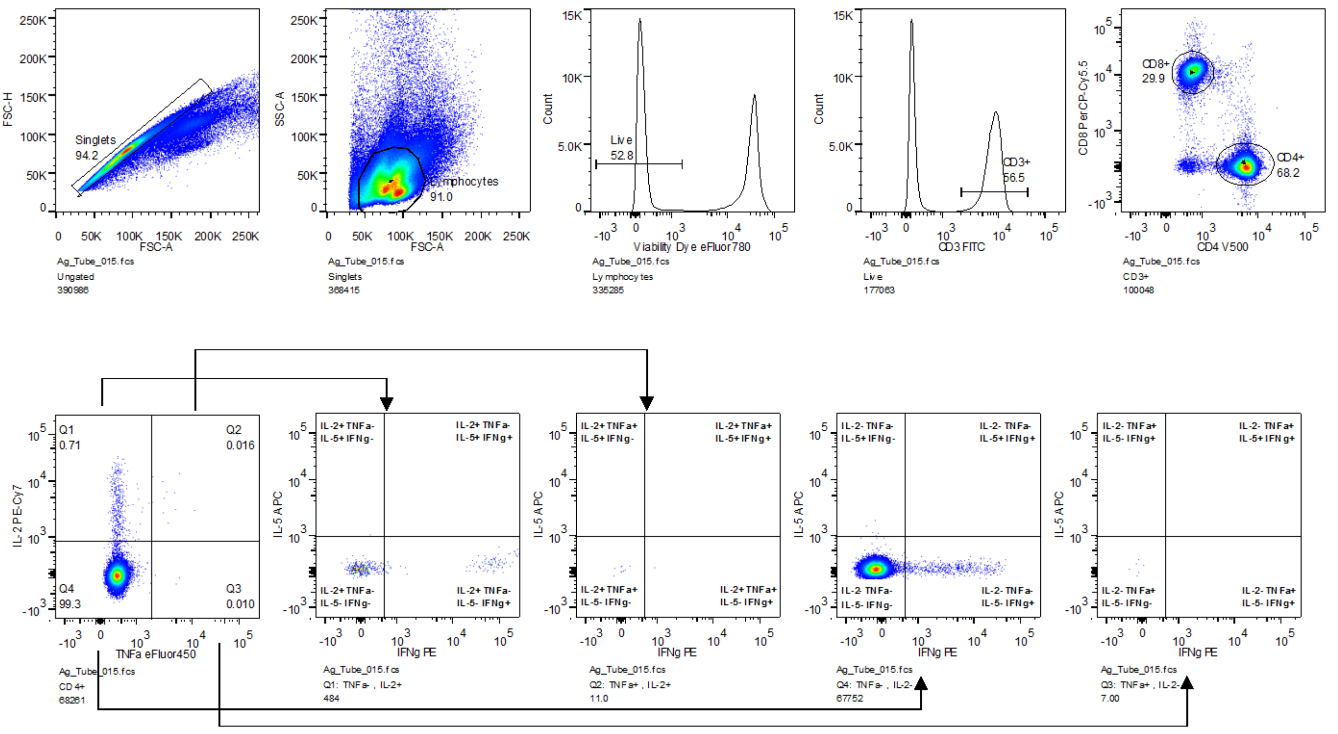

single positive IL-2<br>TNFa- IL-2+ IFNg- IL-5-	double positive IL-2 IL-5<br>TNFa- IL-2+ IFNg- IL-5+	triple positive IL-2 IL-5 IFNg<br>TNFa- IL-2+ IFNg+ IL-5+	quadruple positive<br>TNFa+ IL-2+ IFNg+ IL-5+
single positive TNFa<br>TNFa+ IL-2- IFNg- IL-5-	double positive IL-2 IFNg<br>TNFa- IL-2+ IFNg+ IL-5-	triple positive IL-2 TNFa IL-5<br>TNFa+ IL-2+ IFNg- IL-5+	
single positive IL-5<br>TNFa- IL-2- IFNg- IL-5+	double positive IL-2 TNFa<br>TNFa+ IL-2+ IFNg- IL-5-	triple positive IL-2 TNFa IFNg<br>TNFa+ IL-2+ IFNg+ IL-5-	
single positive IFNg<br>TNFa- IL-2- IFNg+ IL-5-	double positive TNFa IL-5<br>TNFa+ IL-2- IFNg- IL-5+	triple positive TNFa IL-5 IFNg<br>TNFa+ IL-2- IFNg+ IL-5+	
	double positive TNFa IFNg<br>TNFa+ IL-2- IFNg+ IL-5-		
	double positive IL-5 IFNg<br>TNFa- IL-2- IFNg+ IL-5+		
Total single positive	Total double positive	Total triple positive	Quadruple positive
Total responding cells			

Figure S2

Representative flow cytometric dot plots for CD4+ T cells.

Graphical representation of 3 – 5 mice per group is shown in Fig. 5. Splenocytes of immunized mice were stimulated *ex vivo* with A/Uruguay H3N2 split vaccine and co-stimulatory antibodies, then analysed by flow cytometry for CD4+ T cells that produce a combination of IL-2, IL-5, IFNγ or TNFα cytokines by intracellular staining.

Supplemental Figure 2

Representative CD4 plots

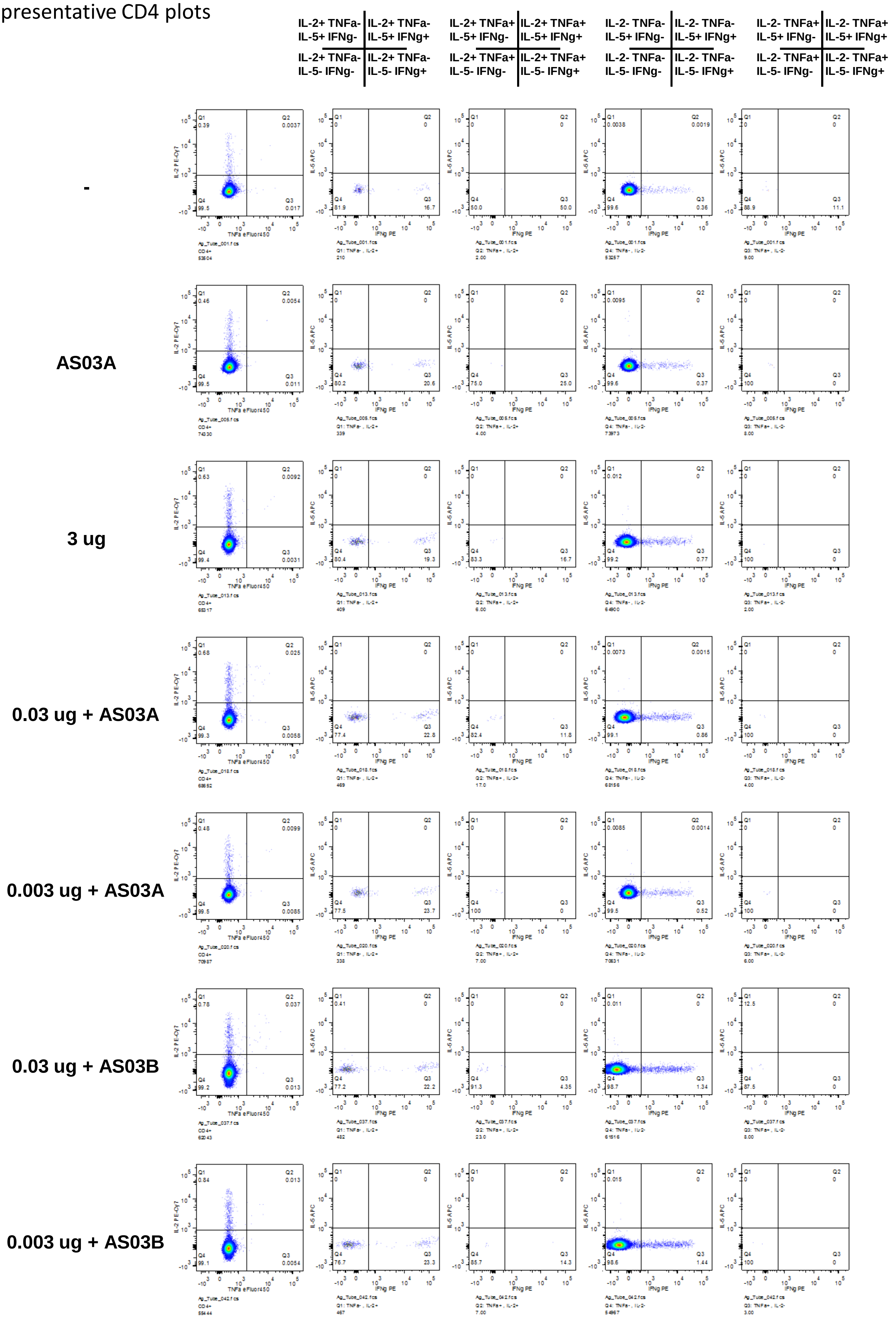

<u>Figure S3</u>

Representative flow cytometric dot plots for CD8+ T cells.

Graphical representation of 3 – 5 mice per group is shown in Fig. 6. Splenocytes of immunized mice were stimulated *ex vivo* with A/Uruguay H3N2 split vaccine and co-stimulatory antibodies, then analysed by flow cytometry for CD8+ T cells that produce a combination of IL-2, IL-5, IFNγ or TNFα cytokines by intracellular staining.

Supplemental Figure 3

Representative CD8 plots

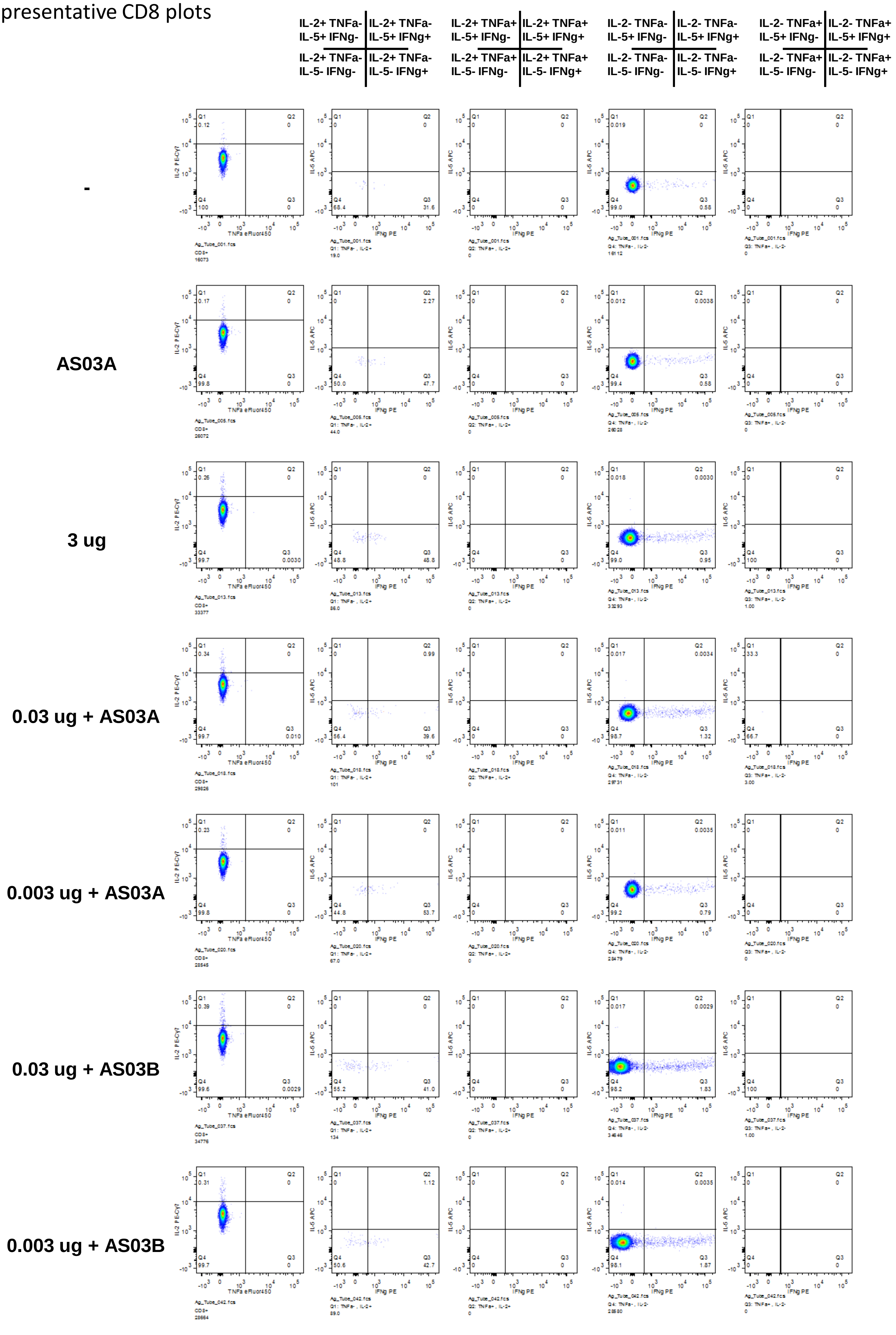

Supplement: Supplementary file 1 [file Data_Sheet1.PDF]
